# Direct measurements of SR free Ca reveal the mechanism underlying the transient effects of RyR potentiation under physiological conditions

**DOI:** 10.1093/cvr/cvu158

**Published:** 2014-06-19

**Authors:** David J. Greensmith, Gina L.J. Galli, Andrew W. Trafford, David A. Eisner

**Affiliations:** Unit of Cardiac Physiology, Institute of Cardiovascular Science, Manchester Academic Health Science Centre, 3.18 Core Technology Facility, 46 Grafton Street, Manchester M13 9NT, UK

**Keywords:** Calcium, Sarcoplasmic reticulum, Ryanodine receptor

## Abstract

**Aims:**

Most of the calcium that activates contraction is released from the sarcoplasmic reticulum (SR) through the ryanodine receptor (RyR). It is controversial whether activators of the RyR produce a maintained increase in the amplitude of the systolic Ca transient. We therefore aimed to examine the effects of activation of the RyR in large animals under conditions designed to be as physiological as possible while simultaneously measuring SR and cytoplasmic Ca.

**Methods and results:**

Experiments were performed on ventricular myocytes from canine and ovine hearts. Cytoplasmic Ca was measured with fluo-3 and SR Ca with mag-fura-2. Application of caffeine resulted in a brief increase in the amplitude of the systolic Ca transient accompanied by an increase of action potential duration. These effects disappeared with a rate constant of ∼3 s^−1^. Similar effects were seen in cells taken from sheep in which heart failure had been induced by rapid pacing. The decrease of Ca transient amplitude was accompanied by a decrease of SR Ca content. During this phase, the maximum (end-diastolic) SR Ca content fell while the minimum systolic increased.

**Conclusions:**

This study shows that, under conditions designed to be as physiological as possible, potentiation of RyR opening has no maintained effect on the systolic Ca transient. This result makes it unlikely that potentiation of the RyR has a maintained role in positive inotropy.

## 1. Introduction

Most of the calcium that activates contraction in the heart comes from the sarcoplasmic reticulum (SR). Calcium is released via the process of calcium-induced calcium release (CICR). On this mechanism, Ca first enters the cell via the L-type Ca channel. Some of this Ca then binds to the SR Ca release channel (ryanodine receptor, RyR), making it open and thereby resulting in the release of a larger amount of Ca (see Bers^1^ for review).

As well as being activated by increased cytoplasmic Ca concentration ([Ca^2+^]_i_), opening of the RyR is sensitive to a variety of other modulators (Meissner^2^ for review). These substances include: phosphorylation,^3^ redox state,^4,5^ *S*-nitrosylation,^6^ and cyclic ADP-ribose.^7^ While it is clear that these agents affect the open probability of the RyR, it is more controversial what the effect will be on the systolic Ca transient.

Phosphorylation of the RyR has been reported to contribute to the positive inotropic effects of β-adrenergic stimulation,^8^ although this has been disputed.^9^ Cyclic ADP-ribose increases the amplitude of the Ca transient,^7^ although it has been suggested that this may be a consequence of stimulation of the sarco-endoplasmic reticulum calcium ATPase (SERCA) rather than the RyR itself,^10^ an explanation disputed by the original authors.^11^

We have previously investigated, in rat ventricular myocytes, the effects of increasing the open probability of the RyR with low concentrations of caffeine. This results in a purely transient increase in the amplitude of the Ca transient. In the steady state, the amplitude of the Ca transient in the presence of caffeine is identical to that in control^12–14^ (see Eisner *et al.*^15,16^ for recent review). The lack of a sustained effect has been suggested to arise from a compensatory decrease of SR Ca content. It is, however, important to note that this reduction of SR Ca content has not been measured directly and a major aim of the present work was, therefore, to address this point.

It has been suggested that our analysis is too simplified and/or not relevant.^17^ For example, it has been argued that our previous work was performed on rats, and that the conclusions may not be relevant to species with longer action potentials where the positive plateau potential will make it harder for the sodium-calcium exchanger (NCX) to pump Ca out of the cell.^11^ Our previous work was performed under voltage clamp, so did not address this issue. We have also been criticized for working at room temperature.^11,18^

It is also important to note that, in our previous work, SR Ca content was measured indirectly, from the integral of the NCX current activated by releasing Ca from the SR. This technique suffers from the disadvantage that it does not provide continuous measurements of SR Ca, which can be compared with changes of systolic Ca. We estimated beat-by-beat changes of SR Ca by convolving measurements of free cytoplasmic Ca with those of Ca buffering and sarcolemmal fluxes. There is, however, a clear need for direct and continuous measurements of SR Ca.

The aim of this paper was to investigate the effects of potentiating Ca release from the SR in species with an action potential similar in shape to that of the human under conditions that are as physiological as possible. For this purpose, we have chosen the sheep and dog, working at body temperature and stimulating the cells to produce normal action potentials rather than using the voltage clamp. We use caffeine as a tool to rapidly and reversibly modulate RyR opening. We have also measured the time course of change in SR Ca content with a low-affinity Ca indicator trapped in the SR. We find that low concentrations of caffeine produce an increase in the amplitude of the Ca transient, which disappears completely within two or three beats. This is accompanied by a decrease of *free* SR Ca, measured directly with an indicator. We conclude that increasing the open probability of the RyR will have no steady-state effect on the amplitude of the Ca transient.

## 2. Methods

Sheep myocytes were isolated at the University of Manchester. Dog myocytes were provided by Astra Zeneca (Alderley Park, Cheshire, UK). All procedures (sheep and dog) accord with the Animals (Scientific Procedures) Act, UK, 1986 and Directive 2010/63/EU of the European Parliament. The experiments were approved by the University of Manchester Ethical Review Board, and the Animal Welfare and Ethics Review Body at Astra Zeneca.

### 2.1 Isolation of ventricular myocytes

Sheep ventricular myocytes were used in some experiments. Female Welsh sheep were killed by an intravenous injection of pentobarbitone (200 mg/kg) and heparin (10 000 i.u.). Once the heart was removed and the ventricles separated, the left descending coronary artery was cannulated and perfused for 10 min with a Ca-free solution containing (in mM): NaCl 134, glucose 11, HEPES 10, 2,3-butandione monoxamine (BDM) 10, KCl 4, MgSO_4_ 1.2, NaH_2_PO_4_ 1.2, and 0.5 mg/mL of bovine serum albumin (BSA), pH 7.34 with NaOH. Tissue digestion was commenced by adding a class 4 collagenase (Worthington, NJ, USA) and protease XIV (Sigma-Aldrich, Gillingham, UK) at typical concentrations of 0.24 and 0.024 mg/mL, respectively, and perfusing for ∼7 min. Following digestion, the ventricles were perfused for 20 min with a taurine solution containing (in mM): NaCl 113, taurine 50, glucose 11, HEPES 10, BDM 10, KCl 4, MgSO_4_ 1.2, NaH­_2_PO_4_ 1.2, CaCl_2_ 0.1, and 0.5 mg/mL of BSA, pH 7.34 with NaOH. Left ventricular mid-myocardial cells were dissociated by gentle agitation. A similar protocol was used to isolate dog ventricular myocytes and has been described in detail previously.^19,20^ Some experiments were performed on cells isolated from sheep where heart failure had been induced by right ventricular tachypacing at 3.5 Hz for 4–6 weeks and confirmed by echocardiography as described previously.^21–23^

### 2.2 Experimental solutions

Cells were superfused with a standard experimental solution containing (in mM): NaCl 140, HEPES 10, glucose 10, KCl 4, probenecid 2, MgCl_2_ 1, CaCl_2_ 1.8, pH 7.35 with NaOH. The probenecid was required to reduce loss of fluorescent indicators from the cell, a particular problem at 37°C_._ Caffeine was added as indicated in the figures.

### 2.3 Measurement of cytoplasmic and SR Ca

In experiments in which only cytoplasmic Ca was measured, cells were loaded with the acetoxymethyl ester (AM) form of either fluo-3 or fura-2 (final concentration 5 and 1 µM, respectively) for 10 min at room temperature. In some experiments (*Figures 6, 7 and S1*), cytoplasmic and SR Ca were measured simultaneously. Here, cells were co-loaded with fluo-3AM (cytoplasmic Ca) and the low-affinity Ca indicator mag-fura-2AM (SR Ca). Cells were incubated (at 37°C) for 30 min with mag-fura-2AM (6 µM) to load the SR. To remove any cytoplasmic component, the cells were re-suspended in a probenecid-free version of the standard experimental solution and incubated (at 37 °C) for a further 30 min. Cells were subsequently loaded with fluo-3 as described above and stored in the probenecid-free solution until use.

Fluo-3 was excited continuously at 488 nm. Where cells were loaded with fura-2 or mag-fura-2, the indicator was excited sequentially at 340 and 380 nm using a monochromator (Cairn Research, Faversham, UK). For both indicators, fluorescence excited at 380 nm decreases with an increasing Ca concentration. At 340 nm, the fluorescence of fura-2 increases with an increasing Ca concentration, but for mag-fura-2 the 340 nm signal is insensitive to Ca. Changes of cytosolic (fura-2) or intra-SR (mag-fura-2) were obtained at 167–250 Hz from the ratio of light excited at 340 : 380. Emitted fluorescence was measured with a 515 nm long-pass filter.

Raw fluorescent signals were calibrated off-line. Background fluorescence was subtracted from all signals. Fluo-3 signals were expressed as *F*/*F*_0_^24^ (where *F*_0_ is the control diastolic level of fluorescence).

To ensure experimental signals were well within the dynamic range of mag-fura-2, following some experiments, we obtained a minimal and maximal fluorescence value. The minimal value was taken as the fluorescence subsequent to SR emptying with 10 mM caffeine.^25^ A maximal florescence value was obtained by applying 10 mM Ca until we observed spontaneous SR Ca release, then applying 1 mM tetracaine to further increase SR Ca (*Figure 6*).^26^

### 2.4 Patch clamp

An Axoclamp 2B voltage-clamp amplifier (Molecular Devices, CA, USA) was used to current or voltage clamp the cells using the perforated patch technique.^27^ Micropipettes (typically 3 MΩ) were filled with a pipette solution containing (in mM): KCl 20, K_3_CH_3_O_3_S 125, NaCl 10, HEPES 10, MgCl_2_ 5, K_2_EGTA 0.1, pH 7.2. Amphotericin B (240 µg/mL, Sigma-Aldrich) was added to provide perforation. Under current clamp, action potentials were triggered using, typically, a 2 ms duration, 1.5 nA current pulse. Under voltage clamp, the Ca current (*I*_Ca-L_) was activated by applying a 100 ms duration step from a −40 mV holding potential to +10 mV. Under voltage clamp, outward currents were inhibited by addition of 4-aminopyridine (5 mM), BaCl_2_ (0.1 mM), and 4,4′-Diisothiocyanatostilbene-2,2′-disulfonic acid (DIDS) (0.1 mM) (Sigma-Aldrich).

### 2.5 Measurement of sarcolemmal Ca fluxes and total SR Ca content

Briefly, under voltage clamp, Ca influx was quantified by integration of the Ca current evoked on depolarization. The NCX (tail) current activated on subsequent repolarization was integrated to calculate Ca efflux.^28^ SR Ca content was quantitatively measured by a rapid application of 10 mM caffeine and integration of the resulting inward NCX current.^25^ We have previously described these manoeuvres in greater detail.^29^

### 2.6 Data analysis and statistics

Data were analysed with custom-written Excel routines.^30^ Statistical significance was determined using either an unpaired *t*-test (to compare two groups) or repeated-measures ANOVA (to compare multiple, sequentially recorded groups).

## 3. Results

**Figure 1:**
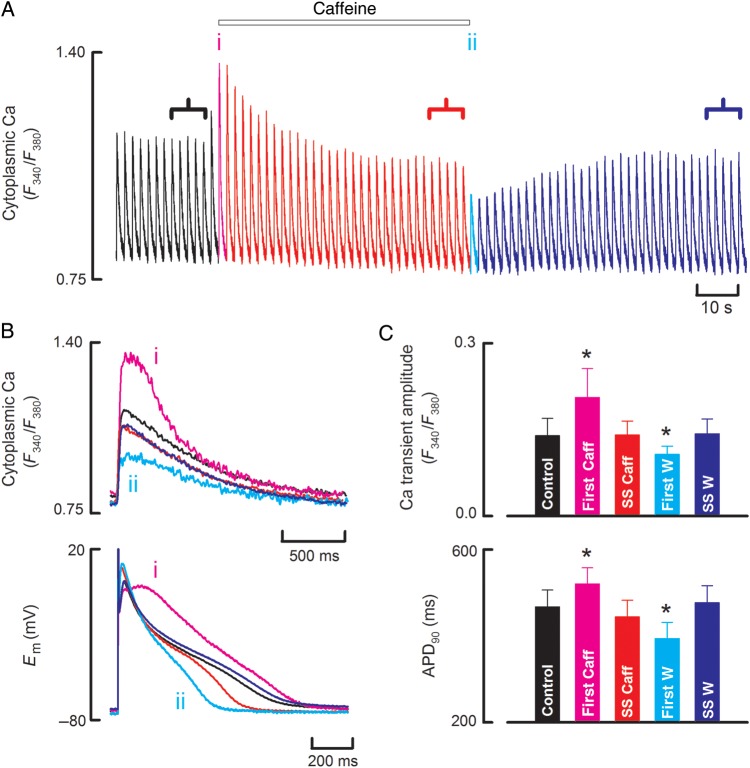
The effects of caffeine on systolic [Ca^2+^]_i_ in sheep ventricular myocytes. (*A*) Time course of changes in cytoplasmic Ca measured with fura-2. Cells were stimulated with current pulses. Caffeine (500 µM) was applied for the period shown. (*B*) Specimen records of Ca (top) and membrane potential (bottom) taken at the points indicated on *A*. (*C*) Average data for Ca transient amplitude (top) and APD (bottom) for (from left to right); control, first beat in caffeine, steady state in caffeine, first beat on wash, steady-state wash. *n* = 12 cells from nine animals. Asterisk represents a statically significant difference.

The experiment illustrated in *Figure 1* (representative of data from 12 cells) was performed on a sheep ventricular myocyte, which was stimulated with depolarizing current clamp pulses to produce action potentials. *Figure 1A* shows that the application of 500 µM caffeine resulted in an increase of systolic [Ca^2+^]_i_ (i) before the amplitude declined to pre-caffeine levels. On removal of caffeine, the amplitude of the Ca transient undershot (ii) before recovering. The amplified records of *Figure 1B* show that the largest Ca transient in caffeine was accompanied by a prolonged action potential. Finally, the mean data of *Figure 1C* show the reproducibility of the increase in amplitude of the Ca transient and action potential duration (APD) on the application of caffeine followed by a recovery to control levels in the steady state with an undershoot to below control after removing caffeine.

**Figure 2:**
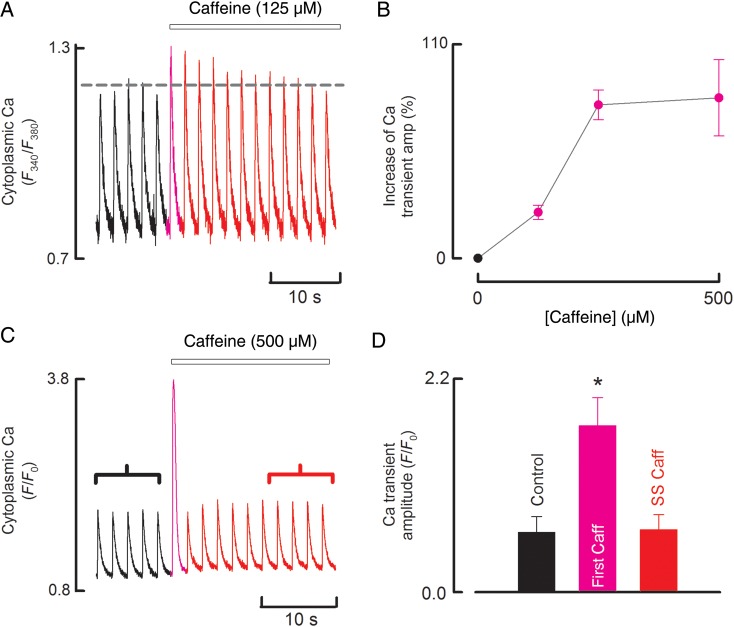
The effects of different caffeine concentrations on systolic [Ca^2+^]_i_ in control and heart failure sheep ventricular myocytes. (*A*) Time course of changes in cytoplasmic Ca measured with fura-2 in control sheep. Cells were stimulated with current pulses. Caffeine (125 µM) was applied for the period shown. (*B*) Mean data showing the concentration-dependence of the increase in the first Ca transient on the application of caffeine *n* = 7 cells from three animals. (*C*) Time course of changes in cytoplasmic Ca measured with fluo-3 in sheep with heart failure. Cells were stimulated with current pulses. Caffeine (500 µM) was applied for the period shown. (*D*) Average data for Ca transient amplitudes in heart failure for (from left to right); control, first beat in caffeine, steady state in caffeine. *n* = 5 cells from three animals. Asterisk represents a statically significant difference.

In subsequent experiments, we investigated the effects of lower concentrations of caffeine. We found that these lower concentrations resulted in qualitatively similar effects as seen for 500 µM. The effects of 125 µM, the lowest concentration which consistently produced a measurable effect, are illustrated in *Figure 2A. Figure 2B* shows the concentration-dependence of the increase in the first Ca transient on the application of caffeine. When the caffeine concentration was increased to 5 mM or above, there was an initial release of Ca from the SR followed by a maintained decrease of the Ca transient (see Supplementary material online, *Figure S2A* and ref.^31^). *Figure S2B* plots the amplitude of the steady-state Ca transient as a function of caffeine concentration. As expected from the data above, at caffeine concentrations up to 500 µM, the amplitude of the systolic Ca transient is constant, but then decreases markedly as the caffeine concentration increases to 5 mM. *Figure 2C* shows the effects of 500 µM caffeine on myocytes taken from a sheep in which heart failure had been induced by rapid ventricular pacing (see Section 2). Onset of heart failure was confirmed by a 55 ± 8% decrease in fractional shortening and a 125 ± 8% increase in end-diastolic internal diameter (for both; *n* = 3, *P* < 0.05), which was comparable to previous findings.^21^ In sheep with heart failure, a transient increase in the amplitude of the Ca transient was also observed (see *Figure 2D* for mean data).

**Figure 3:**
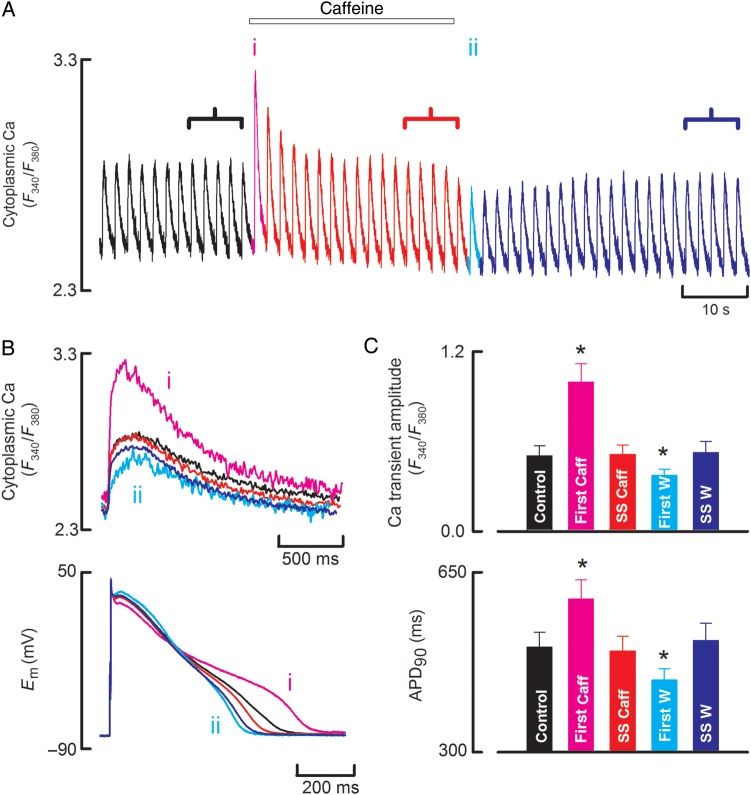
The effects of caffeine on systolic [Ca^2+^]_i_ in canine ventricular myocytes. (*A*) Time course of changes of cytoplasmic Ca measured with fura-2. Cells were stimulated with current pulses. Caffeine (500 µM) was applied for the period shown. (*B*) Specimen records of Ca (top) and membrane potential (bottom) taken at the points indicated on *A*. (*C*) Average data for Ca transient (top) and APD (bottom) for (from left to right); control, first beat in caffeine, steady state in caffeine, first beat on wash, steady-state wash. *n* = 12 cells from four animals. Asterisk represents a statically significant difference.

Subsequent experiments were performed on canine myocytes as (see below) we found that this species was better suited to intra-SR Ca measurements. *Figure 3* shows that the effects of caffeine were very similar to those reported above in the sheep.

**Figure 4:**
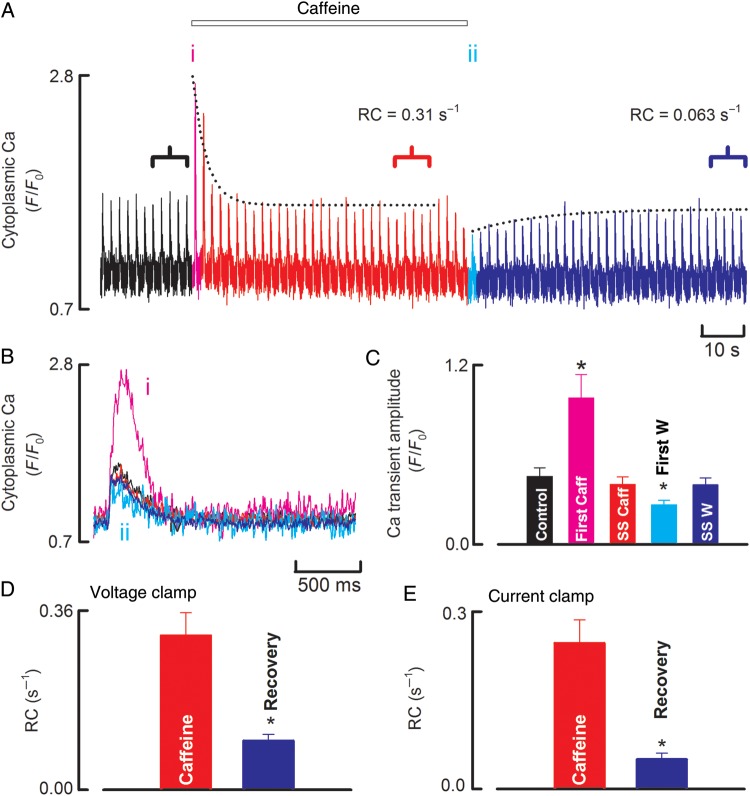
Time course of the onset and recovery of the effects of caffeine in voltage-clamped canine ventricular myocytes. (*A*) Time course of changes in cytoplasmic Ca measured with fluo-3. The cell was stimulated at 0.5 Hz with a 100 ms duration depolarizing pulse from −40 to 10 mV. Caffeine (500 µM) was applied for the period shown. (*B*) Specimen records of Ca obtained at the times shown in *A*. (*C*) Average data (*n* = 10 cells from three animals) for the amplitude of the Ca transient. (*D* and *E*) Rate constant of recovery of the amplitude of the Ca transient under, respectively, voltage and current clamp. In both panels, the left-hand (red) bar is during caffeine and the right-hand (blue) on removal. Asterisk represents a statically significant difference.

The next experiments were performed under voltage clamp to, first, determine whether the effect of caffeine would remain transient, and, secondly, quantify sarcolemmal Ca fluxes. As illustrated in *Figure 4*, the effects of caffeine application on the Ca transient under voltage clamp were similar to those under current clamp. We examined the kinetics of recovery from the effects of both the addition and removal of caffeine. The exponential curves fitted to the peaks of the calcium transients show that both the disappearance of the stimulatory effects of caffeine and the recovery of the undershoot were well fit by a single exponential (*Figure 4A*). As has been previously observed for rat ventricular myocytes,^14^ the rate constant of the exponential is considerably faster during the application of caffeine than on its removal. This is the case whether the data are collected under current or voltage clamp (*Figure 4D* and *E*).

**Figure 5:**
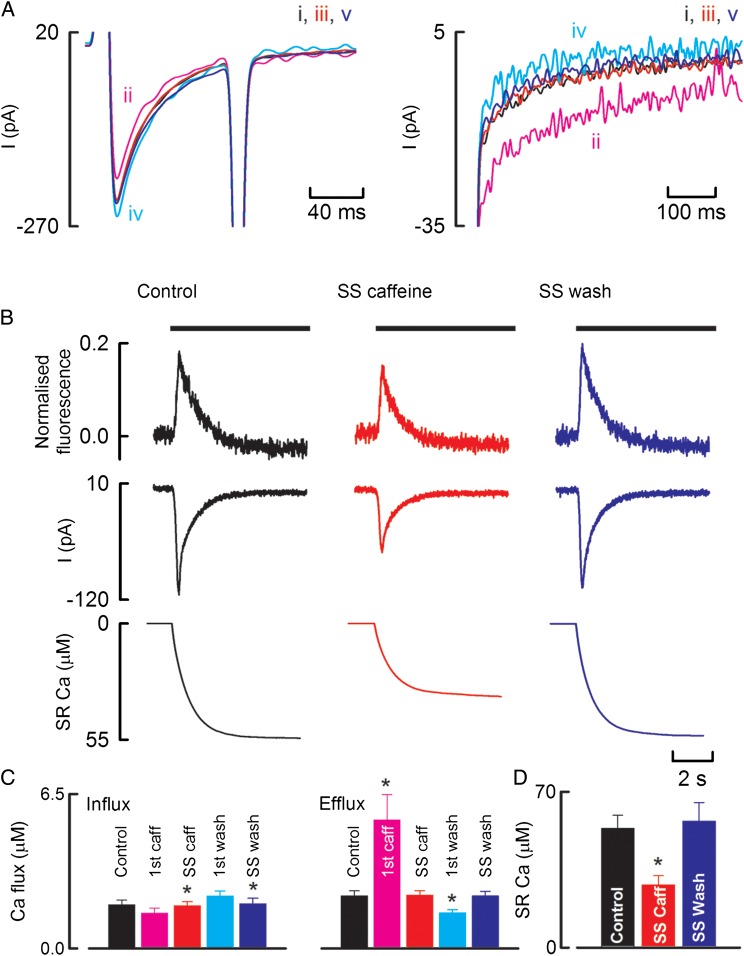
The effects of caffeine on Ca fluxes in voltage-clamped canine ventricular myocytes. (*A*) Membrane currents in response to a 100 ms duration depolarizing pulse from −40 to 10 mV. The right-hand panel shows expanded records of NCX current on repolarization. Traces show (i) control, (ii) first beat in caffeine, (iii) steady-state in caffeine, (iv) first beat in wash, and (v) steady-state wash. (*B*) Measurement of total SR Ca content. Traces show (from top to bottom) Ca, membrane current, integrated current. Caffeine (10 mM) was applied as shown by the horizontal solid bars. The three records were obtained: (from left to right) control, steady state in caffeine, and steady-state wash. (*C*) Average sarcolemmal flux data. The left-hand five bars show influx and the right-hand efflux for six cells from three animals. (*D*) Average measurement of total SR Ca content for five cells from three animals. Asterisk represents a statically significant difference.

The experiment of *Figure 5* was designed to measure the changes of calcium fluxes during the application of caffeine in voltage-clamped canine myocytes. The largest beat in caffeine was accompanied by a decrease in the amplitude of the L-type Ca current and an increase in that of the NCX current. On average (*Figure 5C*), the decrease of Ca entry via the L-type Ca current on the largest beat in caffeine was 21 ± 5% (*n* = 6), whereas the increase of NCX efflux was 145 ± 50% (*n* = 6). In other words, altered NCX makes a much larger contribution to calcium flux balance than does altered Ca entry. These altered Ca fluxes result in decreases of SR Ca content as illustrated by the decrease in the integral of the NCX current in response to the application of 10 mM caffeine (*Figure 5B*). On average (*Figure 5D*), the SR Ca content fell by 47 ± 7% (*n* = 5).

**Figure 6:**
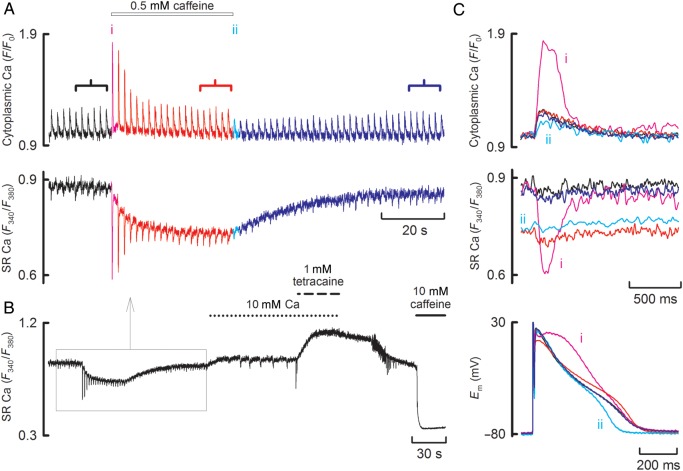
Simultaneous measurement of SR and cytoplasmic Ca. (*A*) Time course traces showing cytoplasmic Ca (top), measured with fluo-3; and SR Ca (bottom) measured with mag-fura-2. (*B*) Time course of the entire experiment. The rectangle shows the period covered by *A*. 10 mM Ca, 1 mM tetracaine, and 10 mM caffeine were applied as shown. (*C*) Specimen data showing (from top to bottom) cytoplasmic Ca, SR Ca, membrane potential.

The measurements of SR Ca content obtained above (*Figure 5B*) were obtained indirectly by applying 10 mM caffeine. This has two drawbacks. First, it measures total as opposed to free SR Ca and, secondly, it does not provide a continuous measure of changes of SR Ca. These problems have been overcome in the data illustrated in *Figure 6A*. Here, the cell was co-loaded with two indicators; in addition to fluo-3, mag-fura-2 was added to measure SR Ca. The fluo-3 trace (*Figure 6A*, top) shows the transient effect of caffeine on systolic Ca. The mag-fura-2 trace (*Figure 6A*, bottom) shows the changes of SR Ca. Under control conditions, decreases of SR Ca accompany the systolic Ca transients. This is shown in greater detail in the expanded records of *Figure 6C*. The initial effect of applying 500 µM caffeine was to increase the SR Ca depletion and prolong the action potential (trace i). In the maintained presence of caffeine, there was a decrease of both the diastolic SR Ca content and the systolic depletion to control levels. On removal of caffeine, both diastolic and systolic SR Ca recovered. The mean data for these experiments are shown in Supplementary material online, *Figure S1*.

One concern with measuring SR Ca content is that the likely free SR Ca may be very close to the level of Ca at which mag-fura-2 saturates. That this is not a major problem is demonstrated in *Figure 6B*, which shows the whole time course of the experiment. Following recovery from caffeine, the external Ca concentration was increased. This produced a modest increase in diastolic SR Ca and spontaneous Ca waves. Subsequent addition of tetracaine to inhibit Ca release from the SR abolished the waves and increased free SR Ca to a level considerably higher than the range encompassed in *Figure 6A*. In other words, the indicator is not near saturation. Some idea of the dynamic range of the indicator is provided by the effects of the subsequent addition of a high concentration (10 mM) of caffeine to decrease SR Ca to low levels. This resulted in a marked decrease in the mag-fura-2 signal.

**Figure 7:**
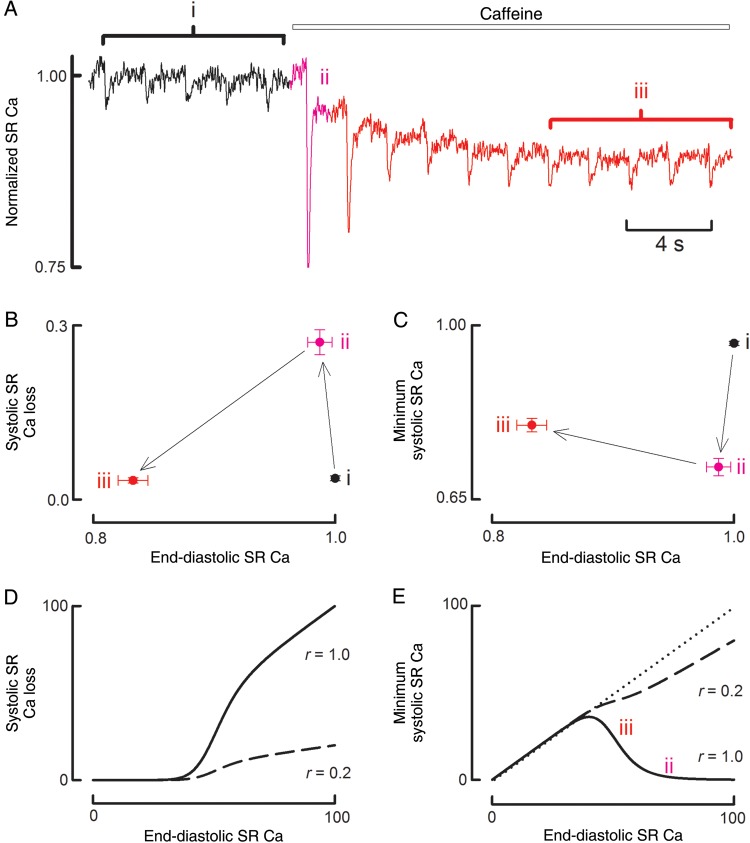
Changes of diastolic and systolic SR Ca content. (*A*) Original data showing SR Ca content measured with mag-fura-2. Caffeine (500 µM) was applied as shown. (*B*) Dependence of the amplitude of the SR systolic depletion on the diastolic SR Ca content. The data points are taken from the regions shown on *A*. To permit comparison between different cells, all fluorescence ratios have been normalized to the diastolic ratio in control. (*C*) Dependence of systolic SR Ca on diastolic SR Ca. The points in *B* and *C* are the mean of 12 cells from six animals. (*D*) Model of the dependence of SR Ca release on diastolic content. The lines show data for *r* = 1 and *r* = 0.2 (see text for details). (*E*) Model of the dependence of systolic SR Ca content on diastolic content. The solid line is for *r* = 1, and the dashed for *r* = 0.2. The dotted line is the line of identity.

The final question we wished to address was whether the decrease of the cytoplasmic Ca transient was entirely explained by a decrease in the filling of the SR (the end-diastolic SR Ca), or whether there was also an effect on the extent to which SR empties of Ca during systole (the minimum systolic SR Ca). Data of *Figure 6A* show that, during the application of caffeine, the decrease of the systolic Ca transient is accompanied by a fall in end-diastolic SR Ca and an increase of minimum systolic SR Ca. This is emphasized in *Figure 7A*. The first beat in caffeine has a low minimum systolic SR Ca (in other words, there is a large depletion of SR Ca). On the next beat, end-diastolic SR Ca has decreased but the minimum systolic level increases. In other words, the decrease in the amplitude of the SR Ca depletion signal is due to a combination of decreased end-diastolic and increased minimum systolic levels. Mean data are shown in *Figure 7B* and *C* for the control, first beat, and steady-state beats in caffeine. *Figure 7B* shows that, as diastolic SR Ca decreases during exposure to caffeine (from ii to iii), there is a decrease of the SR depletion. In other words, amplitude of the SR depletion increases as a function of diastolic SR Ca content. *Figure 7C* shows that minimum systolic SR Ca *increases* as the diastolic level decreases from (ii) to (iii). Therefore, as end-diastolic Ca increases, the SR Ca falls to a lower absolute minimum systolic level.

## 4. Discussion

This paper shows that increasing the open probability of the RyR with caffeine results in only a brief increase in the amplitude of the systolic Ca transient. After a few beats, the Ca transient has the same amplitude as in control. This result is consistent with previous data.^12–14^ There are two main novel findings. (i) The transient nature of modulating RyR open probability on systolic Ca is now seen under conditions designed to be as physiologically relevant to the human situation as possible. Specifically, the experiments were performed at body temperature and we have studied species with long-action potentials (sheep and dog) in contrast to the work on rodents which formed the bulk of the previous work. A similar biphasic response was also found in heart failure. (ii) Using an intra-SR Ca indicator, we show that the transient nature of the response is due to a decrease of SR Ca.

### 4.1 The transient effect of low concentrations of caffeine on Ca transient and APD

When caffeine (at concentrations of 500 µM or below) is applied to current-clamped ventricular myocytes, there is a transient increase in the amplitude of the systolic Ca transient. As described previously, the initial increase in amplitude is due to caffeine increasing the number of RyRs that open and thereby increasing Ca release from the SR. This results in increased Ca efflux and thence a decrease of SR Ca content and the return of the Ca transient amplitude to control levels.^14^ It should also be noted that higher concentrations of caffeine produce a release of Ca from the SR which is independent of electrical stimulation. This depletes the SR of Ca resulting in a maintained decrease in the amplitude of Ca transient. The potentiation of the Ca transient is accompanied by an increase in the action potential duration. However, this increase of APD is not the cause of the increased Ca transient as the latter is observed even when caffeine is applied while stimulating with uniform voltage-clamp pulses. The APD prolongation is likely due to the increased inward NCX current resulting from the larger Ca transient (*Figure 5*). In agreement with work on rat myocytes,^14^ the rate constant of decay of potentiation of the Ca transient during the application of caffeine is considerably faster than that of its recovery on removal of caffeine. We have previously shown that the rate constant of these changes is proportional to the fraction of the SR Ca content that is released on each beat multiplied by the fraction of the Ca transient that is pumped out of the cell (as opposed to being taken back into the SR). Because caffeine increases the fractional release, therefore the rate constant will be greater in the presence of caffeine than on its removal.

### 4.2 Direct measurements of SR Ca content: reciprocal changes of diastolic and systolic SR Ca

A major goal of the present work was to measure change of SR Ca content directly. We have achieved this by using a low-affinity Ca indicator in the SR.^32^ Under control conditions, each systolic Ca transient is accompanied by a decrease of SR Ca content. The addition of caffeine resulted in an immediate increase in the amplitude of the systolic depletion of SR Ca. The amplitude of this depletion then decreased in parallel with the systolic Ca transient. Interestingly, the decrease of SR depletion was a consequence of changes in intra-SR diastolic and systolic Ca. Specifically, while the maximum (end-diastolic) level of intra-SR Ca fell, the minimum systolic level rose. In other words, as diastolic intra-SR Ca falls during the application of caffeine, the amount of depletion decreases by a greater amount such that the minimum systolic SR Ca content *increases*. A similar relationship was predicted by our previous estimates of changes of SR Ca from those of systolic Ca and Ca fluxes,^14^ but, to the best of our knowledge, this is the first direct demonstration of the opposite changes of end-diastolic and minimum systolic SR Ca. The model of *Figure 7D* and *E* shows that this sort of behaviour can be reproduced if the release of Ca from the SR is a steep function of diastolic SR Ca content. On this model, the dependence of the amount of Ca released from the SR on free SR Ca concentration (Ca) is given by:

$$\text{Ca}\;\text{release}=r\times \text{Ca}\times \left[\frac{\text{C}{\text{a}}^{n}}{\text{C}{\text{a}}^{n}+K_{d}^{n}}\right]$$

The fraction of SR Ca that is released is therefore equal to $r\times [\text{C}{\text{a}}^{n}/(\text{C}{\text{a}}^{n}+K_{d}^{n})]$, where *r* is independent of SR Ca. In other words, the fractional release increases with SR Ca with a steepness determined by *n*.

We have arbitrarily assumed a value of 50 µM for *K*_d_ and *n* = 10. In this simple model, we assume that SR Ca buffering is linear. *Figure 7D* shows calculations for two values of *r*. The dashed one represents the situation before adding caffeine with an assumed value of *r* = 0.2. Here, no matter how high SR Ca is, a maximum loss of 20% of the SR Ca occurs. The solid line in *Figure 7D* represents the case in caffeine with *r* = 1.0. Under these conditions, greater Ca release occurs at all SR Ca. Both curves show a similar dependence of Ca release on diastolic SR Ca with one curve having a much greater amplitude than the other. The minimum systolic SR Ca content was then calculated by subtracting the amount released from the end-diastolic content. The resulting curves (*Figure 7E*) show that, for the *r* = 1.0 case (solid line), minimum systolic SR Ca is a biphasic function of SR Ca. The descending limb of the relationship (from points ii to iii) goes in the same direction as the corresponding experimental points in *Figure 7C*. This suggests that the reciprocal changes of diastolic and systolic [Ca^2+^]_i_ can be accounted for by the steep dependence of Ca release on diastolic SR Ca.

### 4.3 The relative contributions of L-type Ca current and NCX to changes of SR Ca

The decay of the caffeine-evoked potentiation of the systolic Ca transient results from a decrease of SR Ca content. This, in turn, is due to increased Ca efflux (on NCX) and decreased Ca entry (via the L-type Ca current). We have described previously how this dependence of sarcolemmal Ca fluxes on SR Ca content ‘autoregulates’ SR Ca. It is clear from the present experiments that the increase of NCX efflux is quantitatively much more significant than is the decrease of Ca influx. On average, on the first beat in caffeine, the efflux increases by 145%, whereas the influx decreases by 19%. It therefore appears that the regulation of SR Ca content depends much more on effects on NCX rather than on the L-type Ca current.

## 5. Conclusions

The results of this paper show that potentiating RyR opening with caffeine does not result in a maintained increase of the Ca transient, even when the experiments are performed under conditions designed to be as physiological as possible. This lack of a maintained effect is due to a directly measured decrease of SR Ca content.

## Supplementary material

Supplementary material is available at *Cardiovascular Research* online.

## Funding

This work was supported by British Heart Foundation grant PG/11/16/28777. Funding to pay the Open Access publication charges for this article was provided by the British Heart Foundation.

## Supplementary Material

Supplementary Data
