# Supplementary material for: Direct measurements of SR free Ca reveal the mechanism underlying the transient effects of RyR potentiation under physiological conditions
Source: Cardiovasc Res. 2014 Jun 19;103(4):554–63. doi: 10.1093/cvr/cvu158 (PMC4145011; doi:10.1093/cvr/cvu158)
Supplement: Supplementary Data [file supp_103_4_554__index.html]

Direct measurements of SR free Ca reveal the mechanism underlying the transient effects of RyR potentiation under physiological conditions — Direct measurements of SR free Ca reveal the mechanism underlying the transient effects of RyR potentiation under physiological conditions — Supplementary Data 

# Direct measurements of SR free Ca reveal the mechanism underlying the transient effects of RyR potentiation under physiological conditions

## Supplementary Data

Supplementary Data

**Files in this Data Supplement:**

- Supplementary Data - Doc file
