# Supplementary material for: Direct measurements of SR free Ca reveal the mechanism underlying the transient effects of RyR potentiation under physiological conditions
Source: Cardiovasc Res. 2014 Jun 19;103(4):554–63. doi: 10.1093/cvr/cvu158 (PMC4145011; doi:10.1093/cvr/cvu158)
Supplement: Supplementary Data [file supp_cvu158_cvu158supp.doc]

**
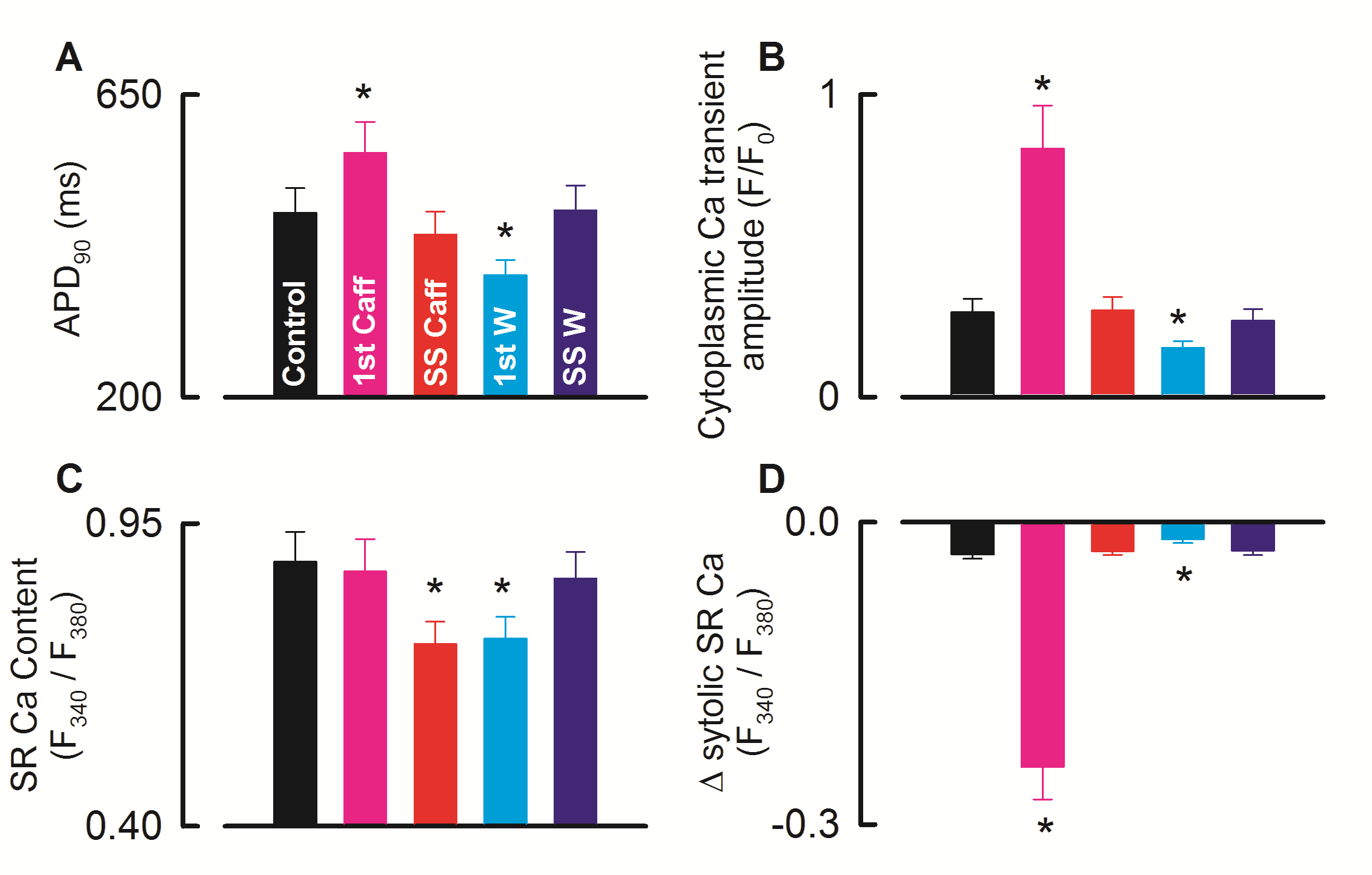
**

**Fig. S1. Average data from experiments in which both cytoplasmic and SR Ca were measured.**

**A**. APD. **B**. Amplitude of cytoplasmic Ca transient. **C**. Diastolic SR Ca. **D**. Change of SR Ca content during systole. Data are a mean of 12 cells from 6 animals.

**
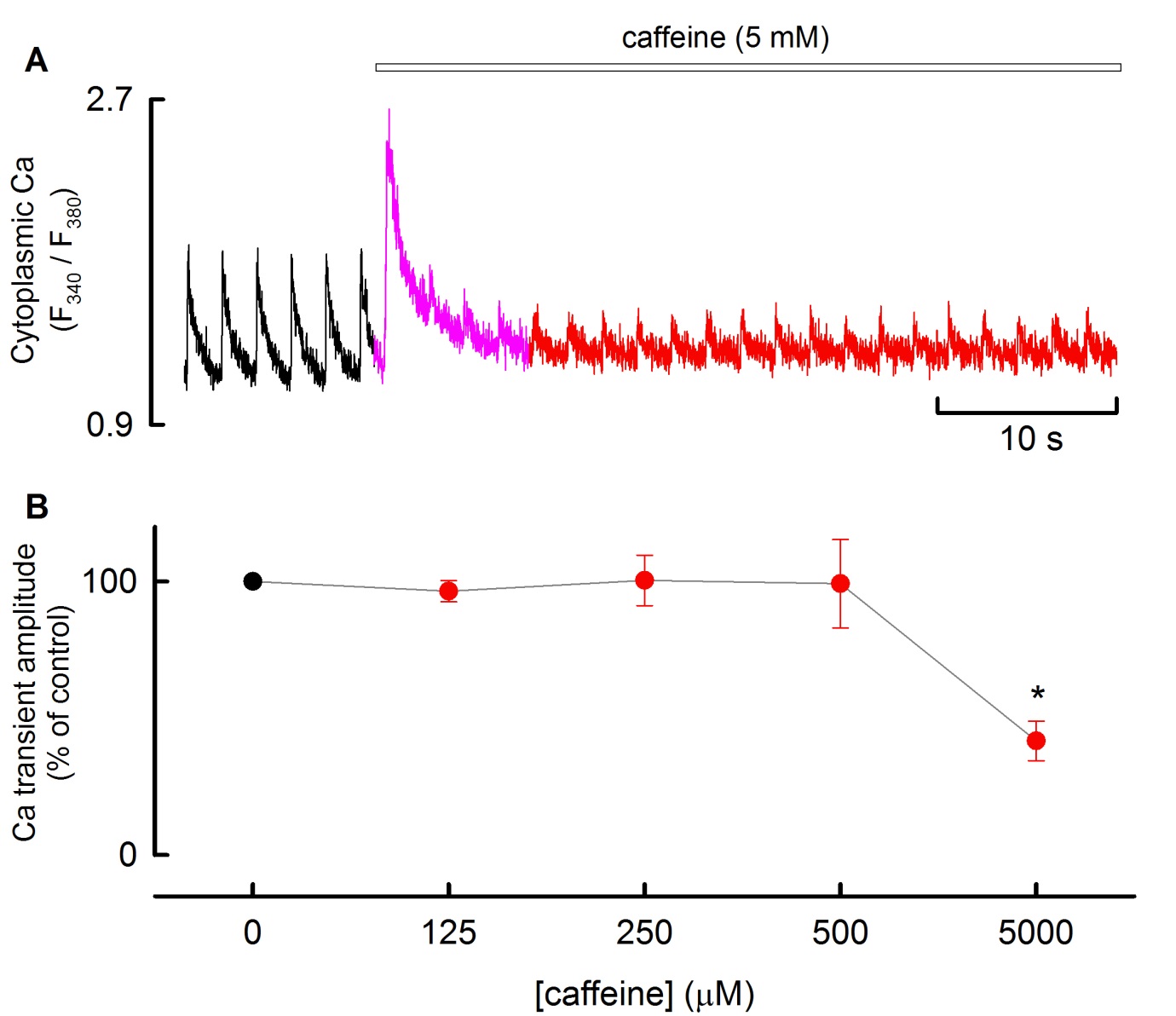
**

**Fig. S2. The effects of caffeine concentration on the steady-state Ca transient. A.** Original data. Cells were stimulated with current pulses and caffeine (5 mM) applied as shown**. B.** Mean data. The Ca transient amplitude (normalized to the value before application of caffeine) is plotted as a function of caffeine concentration. Data from 5-12 cells.
